# Retrospective evaluation of anesthetic techniques in pregnant women with renal transplantation

**DOI:** 10.3906/sag-1905-59

**Published:** 2019-12-16

**Authors:** Asuman SARGIN, Semra KARAMAN, Şeyda CEYLAN, Ali AKDEMİR, İsmet HORTU

**Affiliations:** 1 Department of Anesthesiology and Reanimation, School of Medicine, Ege University, İzmir Turkey; 2 Department of Obstetrics and Gynecology, School of Medicine, Ege University, İzmir Turkey

**Keywords:** Obstetric anesthesia, renal transplantation, pregnancy

## Abstract

**Background/aim:**

The aim of this study was to evaluate anesthesia management in cesarean operation of pregnant women who underwent renal transplantation and the effects on postoperative renal function, retrospectively.

**Materials and methods:**

After obtaining the approval of the ethics committee of our hospital, the records of pregnant women who underwent kidney transplantation and cesarean section between 2007 and 2017 were retrospectively analyzed. The patients’ demographic data, concomitant disease history, the treatment received, and type of anesthesia were retrospectively evaluated and recorded in the follow-up form.

**Results:**

It was found that a total of 47 women who underwent renal transplantation had 47 live births by cesarean section. The mean age of the pregnant women was 30 ± 5.34 years. The mean time between renal transplantation and conception was 95.34 ± 55.02 months. It was found that 14 (29%) of a total of 47 patients had their first pregnancy. The number of patients with a gravidity of 4 and above was 9 (19%). A total of 21 (44.7%) pregnant women had spontaneous miscarriage. Five (10.6%) patients were treated with curettage for therapeutic purposes. Twenty-two (46%) of the patients whose immunosuppressive therapy was continuing were treated with azathioprine, tacrolimus, and prednisolone. The mean gestational age of delivery was 36.5 ± 1.59 weeks. The rate of prepregnancy hypertension diagnosis was 25.5% (n = 12), while the rate of developing gestational hypertension was 21.3% (n = 10). Spinal anesthesia was administered to 42 (91%) of 47 patients who underwent cesarean section. In the preoperative period, the mean value of serum blood urea nitrogen was 62.88 ± 41.97 mg/dL and the mean serum creatinine level was 3.21 ± 6.17 mg/dL. In the postoperative period, these values were 44.4 ± 29.9 mg/dL and 1.91 ± 1.63 mg/dL, respectively. When the pre- and postoperative serum urea and creatinine levels were compared, they were found to be lower in the postoperative period. However, there was no statistically significant difference (P > 0.05). The mean weight of the newborns was determined as 2707.3 ± 501.5 g. While the number of newborns with a low birth weight (<2500 g) was 18 (38%), among them 3 (0.6%) were below 2000 g. It was found that 36.2% (n = 17) of the newborns required intensive care. None of the patients developed graft rejection.

**Conclusion:**

If there is no contraindication, regional anesthesia may be preferred in the first place for pregnant women with renal transplantation. We suggest that this method of anesthesia has some advantages in terms of maintaining postoperative renal function and higher Apgar scores in newborns with low birth weight.

## 1. Introduction

Menstrual cycle disorders and amenorrhea are common problems in women with chronic renal failure. The reason is the suppression of gonadotropin-releasing hormone (GnRH) secretion due to uremia, and hypothalamo-pituitary dysfunction. Especially with new and advanced treatment protocols, the rate of conception is now 0.5%–7% among patients with end stage renal failure. As a result of the increase in the success rate of renal transplantations, renal and endocrine functions are rapidly restored in these patients and ovulatory cycles occur. Although a successful conception is achieved after renal transplantation, numerous maternal and fetal risks may arise. It is important to keep the immunosuppressive drug doses used after renal transplantation in pregnant women who underwent renal transplantation at a proper level in terms of both maintaining the graft function and protecting the health of the mother and fetus. While the number of abortions is quite high in these patient groups, prematurity and intrauterine growth restriction (IUGR) commonly arise in ongoing pregnancies. The anesthetist also has an important role in the management of these patients. With the anesthetic and analgesic techniques to be selected, drugs should not have harmful effects on the patient and the newborn. The literature on anesthetic management of pregnant patients with renal transplantation during cesarean section is quite limited. In this study, we aimed to evaluate and share our patients who underwent cesarean section after renal transplantation in our hospital. 

## 2. Materials and methods

After obtaining the approval of the ethics committee of our hospital, the anesthesia and birth records of 47 pregnant women with kidney transplantation who underwent cesarean section in the Obstetrics and Gynecology Clinic between 2007 and 2017 were analyzed retrospectively. The patients’ demographic data, number and duration of pregnancy, parity, gravity, spontaneous abortion, concomitant disease history, immunosuppressive therapy received, type of anesthesia, duration of anesthesia and operative time, newborn weight, Apgar score, and requirement of intensive care of the mother and the newborn were evaluated retrospectively and recorded in the follow-up form.

For statistical evaluation SPSS 12.0 (SPSS Inc., Chicago, IL, USA) was used. The mean, standard deviation, median, minimum, maximum, frequency, and ratio were used in the definitive statistics of the study and P < 0.05 was accepted as significant. The Wilcoxon test was used to compare urea/creatinine values ​​before and after cesarean section.

## 3. Results 

It was found that a total of 47 women who underwent renal transplantation had 47 live births by cesarean section. The mean age of the pregnant women was 30 ± 5.34 years. The mean time between renal transplantation and conception was 95.34 ± 55.02 months. The number of pregnant women on contraception was 5. Twenty-two (46%) of the patients whose immunosuppressive therapy was continuing were treated with azathioprine, tacrolimus, and prednisolone. The number of patients using cyclosporine, tacrolimus, and prednisolone was 15 (31%). The remaining 10 (21%) patients were using these drugs alone or in different combinations (Table 1).

**Table 1:** Demographic data of patients.

Mean age (years)	30 ± 5.34
Time between transplantation and pregnancy (months)	95.34 ± 55.02
Immunosuppressive therapy Azathioprine + tacrolimus + prednisolone Cyclosporine + tacrolimus + prednisolone Tacrolimus Azathioprine + prednisolone Cyclosporine + prednisolone	22 14 5 2 4

It was found that 14 (29%) of a total of 47 patients had their first pregnancy. The number of patients with a gravidity of 4 and above was 9 (19%). Maternal reasons justified 41 (87.2%) patients undergoing cesarean sections. The median gestational age was 36.5 weeks (min 33–max 39 weeks). It was found that the number of pregnant women with a gestational age below 36 weeks was 10 (21%). Cesarean indications of these patients were due to fetal distress only in 1 pregnant woman. A total of 21 (44.7%) pregnant women had spontaneous miscarriage. Five (10.6%) patients were treated with curettage for therapeutic purposes (Table 2). The rate of prepregnancy hypertension diagnosis was 25.5% (n = 12), while the rate of developing gestational hypertension was 21.3% (n = 10). The number of patients who developed preeclampsia was 6 (12%) (Table 3). Gestational age of these pregnant women was more than 36 weeks. Superimposed preeclampsia was defined as having chronic hypertension with a blood pressure of greater than 140/90 mmHg and proteinuria above 300 mg/L after the 20th week of gestation. None of our patients developed superimposed preeclampsia. During the pregnancy, 4 (0.08%) patients had hydronephrosis. In the preoperative period, the mean serum blood urea nitrogen value was 62.88 ± 41.97 mg/dL and the mean serum creatinine level was 3.21 ± 6.17 mg/dL. In the postoperative period, these values were 44.4 ± 29.9 mg/dL and 1.91 ± 1.63 mg/dL, respectively. When the preoperative and postoperative serum urea and creatinine levels were compared, they were found to be lower in the postoperative period. However, there was no statistically significant difference (P > 0.05).

**Table 2:** Obstetrical data of patients.

	Number of patients (%)
Gestational week ≥36 weeks <36 weeks	36.57 ± 1.59 (min 33–max 39) 37 (78.7%) 10 (21.3%)
Number of gravidity 1 2 3 4 6 7	2.47 ± 1.53 (min 1–max 7) 15 (31.9%) 13 (27.7%) 10 (21.3%) 5 (10.6%) 3 (6.4%) 1 (2.1%)
Parity 0 1 2	0.51 ± 0.658 (min 0–max 2) 27 (57.4%) 16 (34%) 4 (8.5%)
Spontaneous abortion 1 2 3	21 (44.7%) 14 (29.7%) 4 (8.5%) 3 (6.5%)
Cesarean indication* Maternal indication Fetal indication	41 (87.2%) 6 (12.8%)

*Maternal indications included maternal choice, worsening of maternal conditions, unfavorable pelvis, and cephalic-pelvic disproportion. Fetal indications included acute fetal distress.

**Table 3:** Pregnancy-related diseases of patients.

	Number of patients (%)
History of chronic hypertension	12 (25.5%)
Gestational hypertension	10 (21.3%)
Presence of concomitant disease Hydronephrosis Diabetes mellitus Hypothyroidism Hyperthyroidism Heart disease	10 (21.3%) 4 3 1 1 1
Preeclampsia development	6 (12%)

In all patients, standard monitoring (pulse oximetry, noninvasive blood pressure, electrocardiogram) was performed. Spinal anesthesia was administered to 42 (91%) of 47 patients who underwent cesarean section. While only 3 (0.6%) patients received general anesthesia, 2 patients were given general anesthesia due to inadequate spinal anesthesia. One patient refused regional anesthesia. The amount of crystalloids given to the patients in the intraoperative period was 1542.8 ± 535.3 mL. It was determined that only 3 patients received colloid replacement. The intraoperative urine output of the patients was approximately 142.0 ± 42.8 mL. The mean operative time was 61.7 ± 14 min and the mean duration of anesthesia was 78.1 ± 14.2 min. Thiopental and rocuronium were used for induction and sevoflurane, and air was used for maintenance in the patients that received general anesthesia. A total of 9 (19%) patients underwent bilateral tubal ligation upon their own request. 

The mean weight of the newborns was determined as 2707.3 ± 501.5 g. While the number of newborns with a low birth weight (<2500 g) was 18 (38%), only 3 (0.6%) among them were below 2000 g. The mean 1-min Apgar score of the newborns was 7.54 ± 1.70 while the 5-min Apgar score was 8.81 ± 1.63. While the number of newborns with a 1-min Apgar score of ≤7 was 17 (36.2%), the number of those with a 5-min Apgar score of ≤7 was 7 (14.8%). It was found that 36.2% (n = 17) of the newborns required intensive care. None of the newborns had congenital malformation. 

## 4. Discussion 

It has been reported that the rate of pregnancy is 1.5% in women with chronic renal failure who receive long-term dialysis treatment, and the chance of the conception of a woman in reproductive age after transplantation has increased to 5% thanks to the development of the treatment options [1]. In patients with renal transplantation, planning should be done well before pregnancy, and it is recommended to wait about 2 years after transplantation and have minimal or no proteinuria, a serum creatinine level of <180 mol/L or 2 mg/dL, controlled hypertension if present, and no pelvicalyceal distention [2,3]. 

The preoperative examination of the patients is very important for selecting the best anesthetic technique for cesarean section in pregnant women with renal transplantation. It should be focused on concomitant problems and renal functions in the patients, and should be optimized in this respect. 

The American Society of Transplantation recommends that pregnancy should be planned at least 1 year after transplantation in order to obtain the stability of the graft organ [4]. It is stated that this period may be up to 2 years due to the risk of rejection in renal transplant patients [5]. This is necessary to optimize immunosuppressive therapy and to adjust the low dose during pregnancy. Davison [6] examined 1569 pregnancies in 1009 women after renal transplantation, and pregnancy was terminated in 22% of the pregnant women. The rate of spontaneous abortion was 16% and the rate of perinatal mortality was 8%. In all of our patients, the time between transplantation and pregnancy was above 2 years. However, it was found that 19 patients (40%) had more than 3 pregnancies, and most of those previous pregnancies resulted in spontaneous abortion.

Immunosuppressive therapy is necessary for both graft preservation and the prevention of rejection during pregnancy. Gestational diabetes and hypertension are very common in transplantation patients receiving corticosteroid therapy. Patients using glucocorticoids may develop Cushing’s syndrome, and it should be kept in mind that airway management may be challenging [6]. During our study, there were no data suggesting that our patients who were given general anesthesia had difficult intubation. It is necessary to pay attention to the maternal blood pressure, the blood glucose, and the creatinine and electrolytes (potassium, magnesium etc.) in patients who are using calcineurin inhibitors (cyclosporine, tacrolimus) [7]. Interactions of these drugs with anesthetic drugs are also important. For example, as a result of azathioprine use, bone marrow suppression may occur and cause leukopenia or thrombocytopenia [8–11]. The other side effects of azathioprine include gastrointestinal intolerance, liver function abnormalities, and infections. Cyclosporine or tacrolimus may cause reduced gastric emptying and they should be given 4 h preoperatively because of this side effect. The other side effect of cyclosporine is neurotoxicity. Thorough neurological examination is important in patients using cyclosporine as it contributes to seizures, paresthesia, and tremors. Paresthesia is very important if regional anesthesia is preferred. Tacrolimus may interact with many drugs due to its long half-life (57–63 h). In particular, its use with calcineurin inhibitors may potentiate nephrotoxicity [12]. In addition, cremophor, the metabolite of cyclosporine, potentiates the effects of neuromuscular agents [13]. Because of all these reasons, careful examination of the drugs used before anesthesia becomes very important when choosing an anesthetic technique.

A history of chronic hypertension is common in patients with renal transplantation. While the rate of hypertension is 53%–68%, this is mostly due to the hypertensive effect of existing diseases or calcineurin inhibitors before pregnancy [14]. Aggravation of hypertension during pregnancy increases the risk of preeclampsia. While the incidences of preeclampsia during pregnancy vary between 27% and 38%, it has been reported to occur 4 times more in transplantation patients compared to healthy pregnant women [15]. Preeclampsia should be carefully monitored for in renal transplant recipients because its diagnosis is more difficult in these patients, most of who have chronic hypertension and proteinuria [16]. In preeclamptic patients, care should be taken with regard to complications such as fluid balance, hemodynamics, coagulation profile, and airway and pulmonary edema during the preanesthetic assessment. Among our patients, 12 (25.5%) were hypertensive before pregnancy while 10 (21.3%) had gestational hypertension and 6 (12%) had preeclampsia. 

Pregnancy does not cause organ rejection by itself and the transplanted kidney adapts well to the physiological changes associated with pregnancy [17]. Successful pregnancies are seen in patients who do not have high blood pressure or creatinine level, or significant proteinuria without affected graft functions [18]. Hydronephrosis may develop as a result of the growth of the uterus and the compression on the urethra during pregnancy, leading to complications such as reflux nephropathy and pyelonephritis. Only 4 of our patients developed hydronephrosis and none had urinary tract infection.

The first choice in obstetric anesthesia is regional anesthesia, if there is no contraindication. In pregnant women with renal transplantation, anesthesia practice is basically not different from that of other pregnant women, and the patients’ characteristics and physical conditions should be taken into consideration in the decision of general or regional anesthesia [19]. The anesthesia type should be selected depending on the functional status of the transplanted kidney, the cardiovascular status, the hematological status, and the indication for cesarean. In the absence of impaired renal function, anesthesia management is similar to that of a healthy pregnant woman [20,21]. However, although the creatinine levels of patients are normal, the probability of having low glomerular filtration rate (GFR) and plasma flow is high; therefore, the duration of the action of the drugs excreted renally may be longer. From inhalation agents, isoflurane, desflurane, and sevoflurane have been shown to be safe at clinical doses during general anesthesia [22]. Those not excreted renally can be preferred as muscle relaxants (cisatracurium, mivacurium, and atracurium). It should be kept in mind that vecuronium causes renal vasoconstriction. Rocuronium, which is eliminated in the liver, is a muscle relaxant with moderate potency and is a good choice for rapid sequence intubations [21]. In our patients, for general anesthesia, rocuronium was used.

The effect of regional anesthesia on kidney functions depends on the extent and the level of sympathetic block. In hypovolemic patients with impaired cardiac functions, sudden decreases in preload and afterload may decrease the mean arterial pressure, coronary perfusion pressure, and renal perfusion pressure. While renal vascular autoregulation is maintained by regional anesthesia, the perfusion pressure does not change. GFR changes very little due to renal vascular resistance. When regional anesthesia is administered through sensory block of the T6 level, renal blood flow does not change. There are studies indicating the beneficial effects of ephedrine, which is used in the treatment of hypotension during cesarean section, on renal function in major vascular surgeries [23]. Only 8 (17%) of our patients had hypotension, and the mean amount of ephedrine used in symptomatic treatment in addition to fluid replacement was 8.125 mg. Like hypervolemia, hypovolemia should also be avoided in the perioperative period, because transplanted kidneys are very sensitive to hypovolemia [24]. It is thought that this is due to the transplanted kidney being denervated and not be able to rapidly adapt to hypotension with hypovolemia [22]. As a result of this, acute tubular necrosis may arise in the kidney. Therefore, attention should be paid to keeping the urine output above 1 mL/kg/h while hydrating [25]. The amount of crystalloids given to our patients was approximately 1542.8 ± 535.3 mL, while 4 patients received colloid replacement. The intraoperative urine output of the patients was approximately 142.0 ± 42.8 mL. There was not a problem in the urine outputs of the patients in the postoperative period. Especially in the postoperative period, the serum urea and creatinine levels were lower than that of the preoperative period. Although this difference was not statistically significant, we are of the opinion that this may be due to the small size of our sample. In conclusion, spinal anesthesia can be preferred to general anesthesia in terms of maintenance of renal functions. In our study, there was not a change between the pre- and postoperative serum urea and creatinine levels of the patients that were given general anesthesia. 

The rate of preterm birth in pregnant women with transplantation is around 50%. Preterm labor, the premature rupture of membranes, and IUGR are commonly seen in neonates [26]. IUGR has been reported as 26.9% [2,16]. Thompson et al. [27] indicated that the reason for these is associated with immunosuppressive therapy. Oliveira et al. [28] argued that other factors such as hypertension are also associated with this. While 21.3% of our patients had preterm delivery, it was found that the rate of newborns with a weight of 2500 g and below was 38%. The rate of requirement for intensive care was 36.2%.

During pregnancy, renal functions are closely associated with the condition of the fetus [29]. It has been stated that immunosuppressive therapy does not cause a change in the incidence of fetal malformation [30]. None of the newborns in our study had congenital malformation.

In conclusion, pregnant women with renal transplantation are a high-risk group from nephrologic, obstetric, and anesthetic points of view, and maternal and fetal risks are always present. Before deciding on the anesthetic technique, the detailed evaluation of the medical history and physical condition of the mother, as well as not ignoring the condition of the fetus, will be the best approach for the obstetric anesthesiologist. However, we are of the opinion that regional anesthesia is more reliable in terms of preserving the transplanted kidney in patients without contraindication. We suggest that this method of anesthesia has some advantages in terms of maintaining postoperative renal function and higher Apgar scores in newborns with low birth weights.
